# Polypyrrole–Methyl Orange Raman pH Sensor

**DOI:** 10.3390/polym11040715

**Published:** 2019-04-19

**Authors:** Tomasz Czaja, Kamil Wójcik, Maria Grzeszczuk, Roman Szostak

**Affiliations:** Department of Chemistry, University of Wrocław, F. Joliot-Curie 14, 50-383 Wrocław, Poland; tomasz.czaja@chem.uni.wroc.pl (T.C.); kamil.wojcik@chem.uni.wroc.pl (K.W.); maria.grzeszczuk@chem.uni.wroc.pl (M.G.)

**Keywords:** Raman sensor, pH determination, polypyrrole, acid–base indicators, multivariate analysis

## Abstract

An easy-to-prepare pH sensor based on electrochemically obtained polypyrrole doped with methyl orange ions is described. It enables the determination of a pH value in the 3–13 range for volumes below 1 µL. In a wide pH range, resonance and pre-resonance methyl orange Raman spectra, excited with the 514.5 nm line of an Ar^+^ laser, changed noticeably in function of H^+^ concentration. Two types of measurements were performed. In the first case, Raman spectra of the analyzed solutions were collected for samples placed on the sensor surface using a confocal microscope equipped with a 10x objective. Next, measurements were conducted for the same samples without the sensor. On the basis of these spectra, partial least-squares models were elaborated and validated. Relative standard errors of prediction for calibration, validation, and test samples were found to be in the 3.7%–3.9% range. An analogous model build using spectra registered without the sensor was characterized by slightly worse parameters.

## 1. Introduction

One of the most intensively explored areas of analytical chemistry is the design and preparation of optical sensors, which can be used to monitor chemical reactions in different environments. A variety of spectroscopic techniques are used for this purpose. Fluorescence [1,2,3], ultraviolet–visible spectroscopy [4], and surface-enhanced Raman scattering [5,6] are the most popular techniques, while spontaneous and resonance Raman methods are rarely applied [7,8]. The electropolymerization of pyrrole in the presence of an anionic dye, a one-step synthetic procedure, results in the deposition of a conducting polypyrrole–methyl orange (PPy–MO) composite on an electrode surface, which is similar to the reported cases of chemical polymerization of pyrrole [9,10]. The polymerization process preserves the acid–base properties of the dye dopant in the polypyrrole phase. Therefore, the obtained polymer can function as a solid-state platform for quantitative sensing of protons in solutions. Modifications of the dye molecule reflecting changes of the analyte concentration can be monitored using Raman spectroscopy, which is not a very sensitive method. On the other hand, similar information can be obtained more easily from resonance or pre-resonance Raman spectra due to significant enhancement of their intensity [11].

In this study, we describe a pH sensor composed of electrochemically prepared fibres of polypyrrole doped with methyl orange, which enables the determination of a pH value in the 3–13 range for volumes below 1 µL, based on resonance and pre-resonance MO Raman spectra.

## 2. Materials and Methods

### 2.1. Chemicals and Samples

Pyrrole (Sigma-Aldrich, Saint Louis, MO, USA) was distilled under reduced pressure; MO (Alfa Aesar, Karlsruhe, Germany), phosphoric acid (Sigma-Aldrich, Saint Louis, MO, USA), sodium hydroxide (POCH, Gliwice, Poland), and nitrogen 99.9999 % (Air Liquide, Paris, France) were used as supplied. Forty-six samples with pH in the 3–13 range were prepared by titrating a 0.2 M phosphoric acid solution with a 0.2 M sodium hydroxide solution.

### 2.2. Electrochemical Synthesis

**Figure 1:**
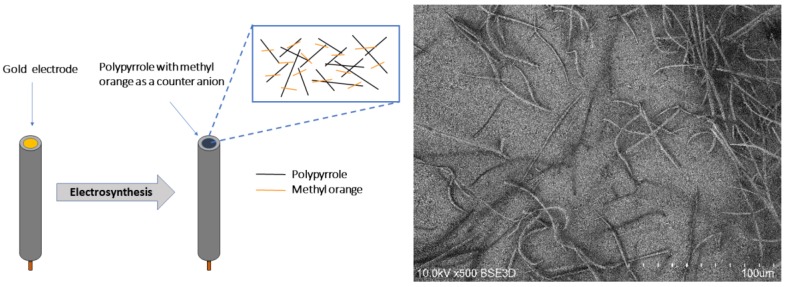
Sensor’s design (left) and SEM image of the sensor surface (right).

An Autolab PGSTAT potentiostat (Metrohm, Utrecht, Netherlands) under GPES (4.8) and FRA (2.4) software control was used for all electrochemical procedures. Cell solutions were deoxygenated by nitrogen flow for 15 minutes and thermostated at 25 °C. A standard three-electrode cell was used. A polycrystalline disc gold electrode without or with deposits was used as the working electrode, and a platinum rod and a saturated silver chloride electrode served as counter and reference electrodes, respectively. MO-doped fibrous polymer was deposited using a potentiostatic method (chronoamperometry) at 0.7 V until a 250 mC/cm^2^ charge had passed in the solution containing 0.15 M pyrrole and 10 mM MO (Figure 1). After electrosynthesis, the resulting polymer electrode was washed with demineralised water.

### 2.3. Surface Morphology

The surface morphology of the PPy–MO films was monitored by a Hitachi S-3400N scanning electron microscope (SEM) (Hitachi, Tokio, Japan) at 10 kV and a back-scattered electron detector.

### 2.4. Spectra

Raman spectra were recorded using a Horiba Jobin Yvon T64000 spectrometer (Horiba Scientific, Kyoto, Japan) equipped with a N_2_-cooled CCD camera, an argon–krypton ion laser operating at 514.5 nm, and a confocal microscope with a 10× objective. The studied solutions were placed on the top of the electrode covered by PPy–MO composite. The scattered radiation, which passed through a notch filter, was analyzed by a spectrograph equipped with a holographic grating with 1800 grooves/mm. Resonance and pre-resonance spectra excited with a power of 5 mW at the sample were recorded in the 800–1800 cm^−1^ range. Electrodes were kept in a glass holder filled with a phosphate-buffered solution.

### 2.5. Chemometrics Models

Partial least-squares (PLS) regression is a popular chemometric tool used for the quantitative modeling of multidimensional spectroscopic data. In this technique, statistically significant factors or latent variables are used to build a regression model between the dependent variables (Y), in our case, the Raman spectra of the samples, and the independent variable (X), which, in this analysis, were the pH values, according to the formula:

Y = XB + E(1)

where B is the matrix of regression coefficients, and E is the error matrix. During the decomposition of the X and Y matrices, the PLS algorithm models the independent and dependent variables maximizing the covariance between the calculated factors. As a result, these matrices can be expressed using the following equations:

X= TP^T^ + E_X_ (2)

Y = UQ^T^ + E_Y_ (3)

where T and U are the scores’ matrices, and P and Q are the loadings. Usually, only the first few factors are retained [12,13].

PLS models were constructed using Nicolet TQ analyst ver. 7 software. The samples were divided into calibration, validation, and test sets. For each sample set, relative standard errors of prediction (RSEP) were calculated according to the equation:

(4) $$RSEP\;\left(\%\right)=\sqrt{\frac{\sum_{i=1}^{n}{\left(X_{i}-X_{i}^{A}\right)}^{2}}{\sum_{i=1}^{n}X_{i}^{A^{2}}}}\;\times \;100\%$$

where *X^A^* is the pH value determined using a pH meter and a glass electrode, *X* is the pH value found from the PLS model, and n is the number of samples. The optimal number of PLS factors was found from the root-mean-square error of cross validation (RMSECV) plots [12,13,14].

## 3. Results

**Figure 2:**
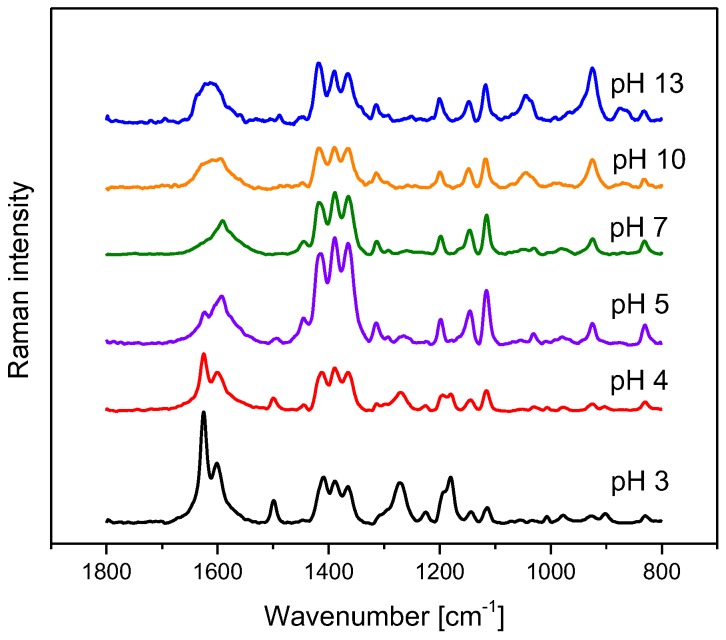
Raman spectra of methyl orange registered at selected pH values.

Methyl orange sodium 4-{[4-(dimethylamino)phenyl]diazenyl}benzene-1-sulfonate is a well-known acid–base indicator that changes colour from red to yellow-orange between 3.1 and 4.4 pH value [15]. In an acidic environment, a strong absorption band in the UV–Vis spectrum with a maximum at 508 nm is observed. Increasing the pH of the solution, causes the appearance of a new band with a maximum at 465 nm [16]. When excited with the 514.5 nm Ar^+^ line, protonated/unprotonated MO species produced resonance and pre-resonance Raman spectra which differed noticeably (Figure 2). In the MO Raman spectra collected at pH = 3, a number of vibrational features connected with the protonated forms of a dye molecule were present, including bands with maxima at 1625, 1499, 1272, and 1181 cm^−1^. Increasing the pH value was associated with the disappearance of these bands and with pronounced changes in the position and shape of some others, for example, at 1604, 1417, 1148, and 1117 cm^−1^. For strongly alkaline samples, bands with maxima at 1046 and 927 cm^−1^ gained intensity. The most visible changes in the recorded Raman spectra were observed at the two extreme pH values, although some modification in the position, intensity, and shape of Raman bands couold be noticed in the whole pH range studied [16,17]. Two types of measurements were performed for the same sample set. In the first case, 100 µL of the prepared solutions was placed on the obtained composite.

**Figure 3:**
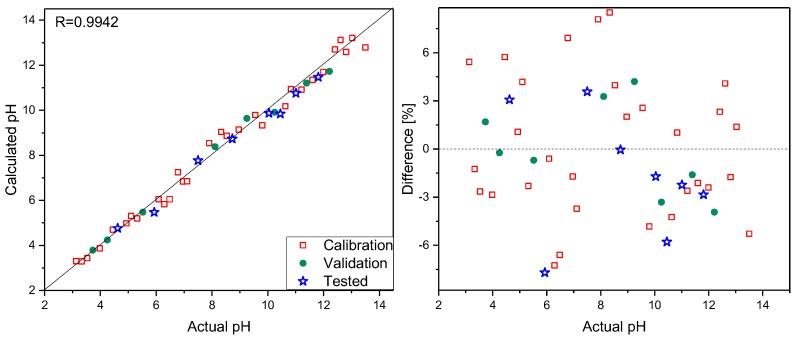
Prediction plot (left) and regression residuals (right) for pH determination based on Raman spectra registered with PPy–MO sensor.

Spectra were recorded focusing the laser beam, using a 10× microscope objective on the sensor surface, and collecting the scattered radiation. Analogous measurements were carried out for the same samples without the PPy–MO sensor. To do so, 10 µL of 0.1% MO water solution was added to 200 µL of each analyzed sample, and Raman spectra were registered under the same objective. To check the possibility of pH determination in small-volume samples, additional measurements were performed on 0.1 µL sample volumes. First, Raman spectra of the composite were recorded, and then a 0.1 µL drop was placed on the sensor surface. The Raman spectra of such small samples strongly resembled the spectra recorded using 100 µL volumes but were less intense. It follows that reliable spectra can be recorded for a drop with a diameter comparable to that of the laser spot on the sensor’s surface. This creates the possibility of pH determination for samples as small as a few nanoliters in volume. Sensor stability was checked by recording several series of spectra of the selected samples within a few weeks. Additionally, spectra were recorded using a sensor that, during the same time period, was not stored in the buffer solution but in air. No spectral changes were observed. On the basis of spectra in the 1130–1685 cm^−1^ range recorded using a PPy–MO sensor, PLS models were elaborated. Thirty samples were used to construct calibration models, eight were selected to validate them, and eight were treated as test samples. The number of latent variables, determined from RMSECV plots, was set to five. The prediction plot and regression residuals for pH determination are shown in Figure 3. The details of the prepared calibration models and the results of the analysis are presented in Table 1. 

As mentioned before, MO was used as a pH indicator in the 3.1–4.4 range. Separate models obtained for low and a high pH ranges of 3.1–7.0 and 7.1–13.50, respectively, were characterized by RSEP errors to be nearly two times smaller than those found for the full-range model. An analogous PLS model was built using spectra registered without a PPy–MO sensor. This model was characterized by slightly worse parameters, and quantification errors were higher compared to those obtained using the PLS model for a PPy–MO sensor (Table 1).

## 4. Conclusions

This report confirms a high potential of the PPy–MO composite for pH determination. The preparation of such a sensor is easy, fast, and inexpensive. In combination with Raman spectroscopy and chemometric methods, it allows to perform an accurate determination of pH values for a wide range for small-volume samples.

## Figures and Tables

**Table 1:** Parameters of the partial least-squares (PLS) models. PPY–MO: polypyrrole–methyl orange, RSEP: relative standard errors of prediction.

Parameter	PPy-MO Sensor	MO in Solution
R ^a^	0.9942	0.9903
R_cv_^b^	0.9724	0.9543
RSEP calibration	3.91	5.11
RSEP validation	3.68	5.37
RSEP test	3.66	4.94
Number of PLS factors	5	5

^a^ correlation coefficient, ^b^ correlation coefficient of cross validation
